# ASK1 inhibitor treatment suppresses p38/JNK signalling with reduced kidney inflammation and fibrosis in rat crescentic glomerulonephritis

**DOI:** 10.1111/jcmm.13705

**Published:** 2018-07-11

**Authors:** Liv A. Amos, Frank Y. Ma, Greg H. Tesch, John T. Liles, David G. Breckenridge, David J. Nikolic‐Paterson, Yingjie Han

**Affiliations:** ^1^ Department of Nephrology Monash Medical Centre Clayton Vic. 3168 Australia; ^2^ Monash University Centre for Inflammatory Diseases Monash Medical Centre Clayton Vic. 3168 Australia; ^3^ Gilead Sciences Inc. Foster City CA USA

**Keywords:** ASK1, inflammation, JNK, macrophage, p38 MAPK, thrombosis

## Abstract

Activation of p38 mitogen‐activated protein kinase (MAPK) and c‐Jun amino terminal kinase (JNK) is prominent in human crescentic glomerulonephritis. p38 and JNK inhibitors suppress crescentic disease in animal models; however, the upstream mechanisms inducing activation of these kinases in crescentic glomerulonephritis are unknown. We investigated the hypothesis that apoptosis signal‐regulating kinase 1 (ASK1/MAP3K5) promote p38/JNK activation and renal injury in models of nephrotoxic serum nephritis (NTN); acute glomerular injury in SD rats, and crescentic disease in WKY rats. Treatment with the selective ASK1 inhibitor, GS‐444217 or vehicle began 1 hour before nephrotoxic serum injection and continued until animals were killed on day 1 (SD rats) or 14 (WKY rats). NTN resulted in phosphorylation (activation) of p38 and c‐Jun in both models which was substantially reduced by ASK1 inhibitor treatment. In SD rats, GS‐444217 prevented proteinuria and glomerular thrombosis with suppression of macrophage activation on day 1 NTN. In WKY rats, GS‐444217 reduced crescent formation, prevented renal impairment and reduced proteinuria on day 14 NTN. Macrophage activation, T‐cell infiltration and renal fibrosis were also reduced by GS‐444217. In conclusion, GS‐444217 treatment inhibited p38/JNK activation and development of renal injury in rat NTN. ASK1 inhibitors may have therapeutic potential in rapidly progressive glomerulonephritis.

## 1 INTRODUCTION

There are two main stress‐activated protein kinases, the p38 mitogen‐activated protein kinase (MAPK) and the c‐Jun amino terminal kinase (JNK).^1^ These enzymes are expressed in most cell types and can be activated by a wide range of stresses such as reactive oxygen species, osmotic stress, hyperglycaemia, growth factors, pro‐inflammatory cytokines and Toll‐like receptor activation.^2^ Activation of p38 and JNK signalling promotes the inflammatory response and cell death.^3^

Compared to the low basal level of p38 and JNK signalling in the normal kidney, there is increased activation of the p38 and JNK pathways in many cells types in human kidney disease which is most pronounced in rapidly progressive crescentic glomerulonephritis.^4^, ^5^, ^6^, ^7^ This increased p38 and JNK activation is evident in infiltrating cells as well as intrinsic kidney cells, including podocytes, mesangial cells, tubular epithelial cells and fibroblasts.^4^, ^5^, ^6^, ^7^ Activation of p38 signalling correlates with renal function across a range of glomerulonephridities, with glomerular p38 activation correlating with segmental proliferative and necrotic lesions and interstitial p38 activation correlating with interstitial inflammation and fibrosis.^7^ Similarly, across a range of glomerular diseases, JNK activation correlates with glomerulosclerosis, while JNK signalling in the tubulointerstitial compartment correlates with macrophage infiltration, interstitial fibrosis and renal dysfunction.^5^, ^6^

Small molecule inhibitors of p38 MAPK can inhibit inflammation and kidney damage in models of antiglomerular basement membrane (GBM) glomerulonephritis.^8^, ^9^, ^10^, ^11^ Subsequently, p38 inhibitors have been shown to suppress acute kidney injury, renal inflammation and renal fibrosis across a range of animal models.^12^, ^13^, ^14^, ^15^ Similarly, small molecule JNK inhibitors can suppress renal injury in several disease models, including crescentic glomerulonephritis^16^, ^17^, ^18^ However, clinical trials evaluating p38 and JNK inhibitors in human inflammatory and fibrotic disorders have failed because of lack of efficacy or side effects such as liver toxicity.^19^, ^20^ Therefore, therapeutic strategies that reduce the activation of the p38 and JNK pathways during pathologic stress, while sparing the physiological roles of these enzymes, may lead to greater efficacy and a better risk/benefit profile.

The p38 and JNK enzymes are activated by a cascade of phosphorylation reactions performed by members of the MAPKKK (MAP3K) and MKK enzyme families. There are more than 20 members of the MAP3K family, many of which have the ability to activate the downstream p38 and JNK enzymes in cell culture studies, although few members of this family have been show to activate p38 and JNK in vivo.^21^ We have investigated the role of one of these enzymes, apoptosis signal‐regulating kinase 1 (ASK1/MAP3K5), as a mechanism of p38/JNK activation in kidney disease based on several reasons. First, ASK1 is kept inactive under normal physiological conditions through binding to the reduced form of thioredoxin and only undergoes auto‐activation upon the oxidation and dissociation of thioredoxin during pathological oxidative stress—an important feature in many forms of kidney disease.^3^, ^22^ Second, mice lacking the *Ask1* gene have a normal phenotype consistent with a lack of involvement of ASK1 in normal physiology.^3^, ^22^ Third, p38 and JNK are the only known downstream targets of ASK1 activation thereby providing a potentially selective means to target these pathways.^3^, ^22^

Using *Ask1* gene‐deficient mice, we have shown that activation of p38 MAPK, and to a lesser extent JNK, is dependent upon ASK1 in the obstructed kidney, while administration of a highly selective ASK1 inhibitor (GS‐444217) in experimental diabetic kidney disease also suppressed p38 and JNK activation.^23^, ^24^ However, it is unknown whether p38 and/or JNK activation depends upon ASK1 in the aggressive renal inflammation seen in crescentic glomerulonephritis. Indeed, the answer to this question is not obvious as ASK1 is not required for acute IL‐1 or LPS‐induced p38 activation in cultured tubular epithelial cells,^23^ and IL‐1 contributes to the development of crescentic disease,^25^ while LPS exacerbates glomerular injury.^26^ This study used the highly selective ASK1 inhibitor, GS‐444217, to ask whether: (i) an ASK1 inhibitor can suppress p38 and JNK activation in crescentic glomerulonephritis, and (ii) an ASK1 inhibitor can suppress disease development. We addressed these questions in two well characterized models of nephrotoxic serum nephritis in different rat strains: acute (day 1) glomerular inflammation in outbred Sprague‐Dawley (SD) rats and progressive (day 14) crescentic disease in inbred Wistar Kyoto (WKY) rats.

## 2 MATERIALS AND METHODS

### 2.1 Reagents

Mouse monoclonal antibodies included; ED1 (CD68, macrophages) and R73 (rat αβ T‐cell receptor) (Serotec, Oxford, UK), RP1 (neutrophils) (Becton Dickinson, San Diego, USA) and anti‐α‐tubulin (Abcam, Cambridge, UK). Rabbit polyclonal antibodies included anti‐phospho‐p38 Thr180/Tyr182 and anti‐phospho‐c‐Jun Ser63 (Cell Signaling, Boston, MA, USA) and goat anti‐γ‐fibrinogen (Santa Cruz biotechnology, CA, USA). Biotinylated antibodies included goat anti‐mouse IgG and goat anti‐rabbit IgG (Zymed, San Francisco, CA, USA). Immunofluorescence staining used FITC (Fluorescein isothiocyanate)‐conjugated rabbit polyclonal antibodies against sheep IgG Dako, Glostrup, Denmark, rat IgG (Sigma‐Aldrich, Castle Hill, NSW, Australia) and rat C3 (Cappel, Malvern, PA, USA).

### 2.2 ASK1 inhibitor

GS‐444217 was synthesized by Gilead Sciences in Foster City, CA.^24^ GS‐444217 inhibits ASK1 kinase activity in vitro with an IC_50_ of 2.87 nmol/L. In a kinase selectivity panel (KINOMEscan; DiscoverRX Corporation, Fremont, CA), GS‐444217 exhibited 50‐fold greater affinity for ASK1 compared to all of the other 451 kinases measured.

### 2.3 Rat models of nephrotoxic serum nephritis (NTN)

Outbred female Sprague‐Dawley (SD) rats (160‐180 g) were obtained from Monash Animal Services. Inbred female Wistar Kyoto (WKY, 160‐190 g) were obtained from the Animal Resources Centre (Perth, WA, Australia).

Acute NTN was induced in SD rats by flank immunization with sheep IgG in Freund's complete adjuvant followed 5 days later (day 0) by tail vein injection of sheep anti‐rat GBM serum (5 mL/kg). Animals were killed 24 hours later. Urine was collected during this 24‐hour period. Groups of 6 rats received twice daily oral gavage with GS‐444217 (30 mg/kg) or vehicle alone (5:10:10:75 ratio of ethanol; distilled water; solutol HS‐15; propylene glycol), starting 1 hour before anti‐GBM serum injection with the last gavage 1 hour before being killed. Peak serum drug levels occur approximately 1 hour postgavage. Serum levels of GS‐444217 were 24.6 ± 21.6 μmol/L (Cmax) at the time of killing animals. This peak level provides >EC_90_ coverage of the target and has excellent selectivity for ASK1 in cell‐based assays. SD rats without experimentation were used as the normal controls.

Crescentic NTN was induced in WKY rats by flank immunization with sheep IgG in Freund's complete adjuvant followed 7 days later (day 0) by tail vein injection of sheep anti‐rat GBM serum (5 mL/kg). Animals were killed 14 days later. Groups of 8 rats received twice daily oral gavage, starting 1 hour before anti‐GBM serum injection, with GS‐444217 (30 mg/kg) or vehicle alone. WKY rats without experimentation were used as the normal controls. Serum levels of GS‐444217 were 26.6 ± 21.6 μmol/L at the time of killing animals on day 14 NTN. Urine collections were performed on days 1, 7 and 13. All animal experimentation was approved by the Monash Medical Centre Animal Ethics Committee and was performed in accordance with Australian National Health and Medical Research Council guidelines for animal experimentation.

Serum and urine creatinine were measured by the Department of Biochemistry (Monash Health) using the Jaffe reaction with alkaline picrate. Total protein levels were measured in urine samples by a Coomassie Protein Assay Kit (ThermoFisher Scientific, Rockland, IL).

### 2.4 Pathology

Glomerular pathology was examined on periodic acid Schiff (PAS)‐stained sections. On day 1 NTN, the percentage of glomeruli exhibiting capillary thrombosis was scored in 50 glomerular cross sections (gcs) per animal. On day 14, the percentage of glomeruli exhibiting crescent formation were scored in 50 gcs per animal. Scoring was performed on blinded slides.

### 2.5 Immunostaining and quantification

Immunoperoxidase staining for CD68+ macrophages and fibrinogen was performed on formalin‐fixed paraffin sections. Antigen retrieval using 10 mmol/L sodium citrate was followed by an avidin–biotin peroxidase complex staining method as previously described.^27^, ^28^ Immunoperoxidase staining for RP1+ neutrophils and R73+ T cells was performed on frozen sections of tissue fixed in 2% paraformaldehyde–lysine–periodate. Immunofluorescence staining for glomerular deposition of sheep IgG, rat IgG and rat C3 was performed on snap frozen tissues.

The number of glomerular ED1+ macrophages, RP1+ neutrophils and R73+ T cells was counted in at least 50 gcs per animal under high power (×400). Interstitial ED1+ macrophages and R73+ T cells were counted in medium power fields (×250) covering >90% of the cortex. Glomerular fibrinogen staining was scored in a semi‐quantitative fashion as follows: 0, no staining; 1+, staining in <25% of the glomerulus; 2+, 25%‐50% staining, and; 3+, >50% staining. All scoring was performed on blinded slides.

### 2.6 Real time RT‐PCR

A section of kidney cortex was snap frozen and stored at −80°C until use. RNA was extracted from frozen tissues using the RiboPure RNA isolation kit (Ambion, Austin,TX, USA). RNA was reverse transcribed with random hexamer primers and the Superscript II kit (Invitrogen, Carlsbad, CA, USA). Real‐time RT‐PCR was performed on a StepOne machine (Applied Biosystems, Mulgrave, Vic., Australia) as previously described.^17^, ^29^ Probes and primers for detection of TNF‐α, NOS2, KIM1, CD68, MCP‐1, MMP‐12, CD206, FoxP3, Col I, α‐SMA, TGF‐β1 have been described previously.^17^, ^29^ Primers and probes for MMP‐9, CD3ε, IL‐2, RANTES and 18S ribosomal RNA were purchased from Applied Biosystems. All amplicons were normalized against the 18S RNA internal control (Applied Biosystems). The relative amount of individual mRNA species was calculated using the comparative Ct method.

### 2.7 Western blotting

A section of kidney cortex was snap‐frozen and stored at −80°C until use. Frozen kidney samples were homogenized in RIPA lysis buffer as previously described.^30^ Samples were run on 12% SDS‐PAGE gels and then electro‐blotted on to nitrocellulose membranes. Labelled protein bands were detected using the Odyssey Infrared Image Detection system (LICOR). Detection of α‐tubulin was used as the loading control. Densitometry analysis was performed as described. The Gel Pro Analyzer program (Media Cybernetics) was used for densitometry analysis.

### 2.8 Statistical analysis

Data are presented as mean ± standard deviation. Analysis was performed by ANOVA using Tukey's or the Kruskal‐Wallis post‐test for multiple comparisons. Analyses were performed using GraphPad Prism 6.0 software (San Diego, CA, USA).

## 3 RESULTS

### 3.1 GS‐444217 inhibits glomerular injury in acute NTN in SD rats

**Figure 1:**
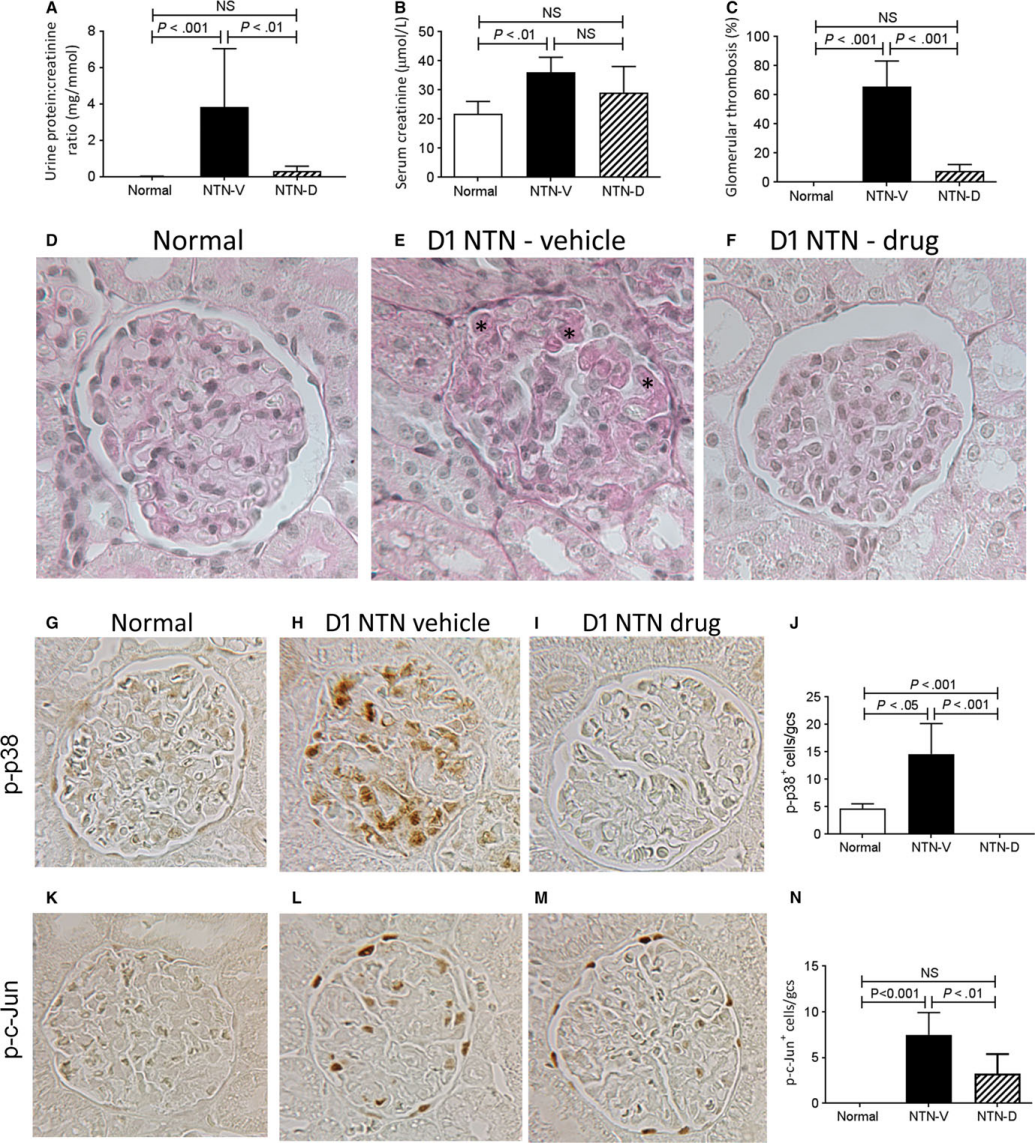
Glomerular injury and activation of p38 and c‐Jun amino terminal kinase (JNK) in day 1 nephrotoxic serum nephritis (NTN). Disease was induced in groups of 6 SD rats which were treated with GS‐444217 (drug; NTN‐D) or vehicle (NTN‐V) alone. A group of 6 normal rats served as control. A, urine total protein:creatinine ratio, (B) serum creatinine, (C) percentage of glomeruli exhibiting thrombosis in periodic acid Schiff (PAS) stained sections. D‐F, PAS stained sections: D, normal rat kidney; E, vehicle‐treated disease exhibiting capillary thrombosis (*); F, drug treated disease. G‐I, immunostaining for phosphorylated p38 (p‐p38): G, weak staining for p‐p38 in some glomerular cells in normal rat kidney; H, vehicle‐treated day 1 NTN (NTN‐V) shows many p‐p38+ cells in the glomerular tuft; I, GS‐444217 (drug; NTN‐D) treatment abolishes p‐p38 staining in day 1 NTN. J, Quantification of p‐p38+ cells within the glomerular tuft. K‐M, immunostaining for phosphorylated c‐Jun^Ser63^ (p‐c‐Jun): K, No staining in normal rat kidney; (L) p‐c‐Jun+ cells are seen in the glomerular tuft and Bowman's capsule in vehicle‐treated day 1 NTN; M, GS‐444217 (drug) treatment reduces the number of p‐c‐Jun+ cells in the glomerular tuft in day 1 NTN. N, Quantification of p‐c‐Jun+ cells within the glomerular tuft. Original magnification, ×400 (D‐F, G‐I, K‐M). Data are mean ± SD. Analysed by one‐way ANOVA with Tukey's multiple comparison test

Acute nephrotoxic serum nephritis (NTN) was induced in outbred Sprague‐Dawley rats. This model features acute glomerular inflammation with glomerular injury as shown by increased protein excretion in the urine.^10^, ^16^ Consistent with previous studies, vehicle‐treated rats exhibited proteinuria, glomerular thrombosis and an acute reduction in renal function on day 1 NTN (Figure 1A‐E). Drug treatment largely prevented the development of proteinuria and glomerular thrombosis, with a trend towards improved renal function (Figure 1A‐F).

Given the lack of commercial antibodies suitable for detecting the activated form of ASK1, we examined phosphorylation (activation) of p38 and c‐Jun; the downstream targets of ASK1 activation. Prominent activation of both p38 and JNK pathways was evident in glomerular cells in vehicle treated rats on day 1 NTN (Figure 1G‐N). Drug treatment abrogated p38 signalling and caused a partial reduction of JNK activation in glomerular cells (Figure 1G‐N).

**Figure 2:**
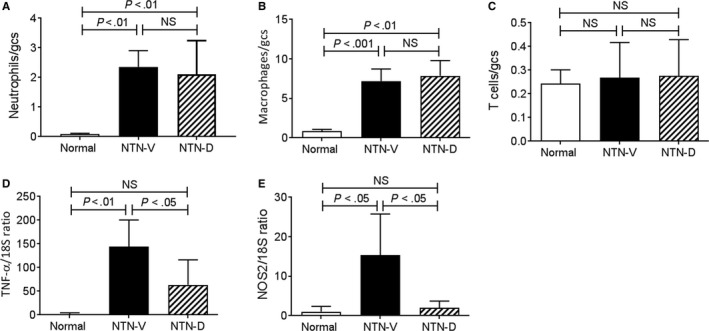
Glomerular leucocyte recruitment and inflammation in day 1 nephrotoxic serum nephritis (NTN). Disease was induced in groups of 6 SD rats which were treated with GS‐444217 (drug; NTN‐D) or vehicle (NTN‐V) alone. A group of 6 normal rats served as control. A, Glomerular neutrophils. B, Glomerular macrophages. C, Glomerular T cells. Glomeruli were isolated for RT‐PCR analysis of: D, TNF‐α, and (E) NOS2. Data are mean ± SD. Analysis by one‐way ANOVA with Tukey's multiple comparison test

Analysis of glomerular leucocytes found that drug treatment did not affect glomerular infiltration of macrophages and neutrophils on day 1 NTN, while no glomerular T‐cell infiltration was evident in either group at this time‐point (Figure 2A‐C). Despite the lack of effect upon myeloid cell recruitment, drug treatment substantially reduced glomerular expression of the pro‐inflammatory molecules; TNF‐α and NOS2 (Figure 2D,E).

### 3.2 GS‐444217 inhibits the development of crescentic disease in progressive NTN in WKY rats

**Figure 3:**
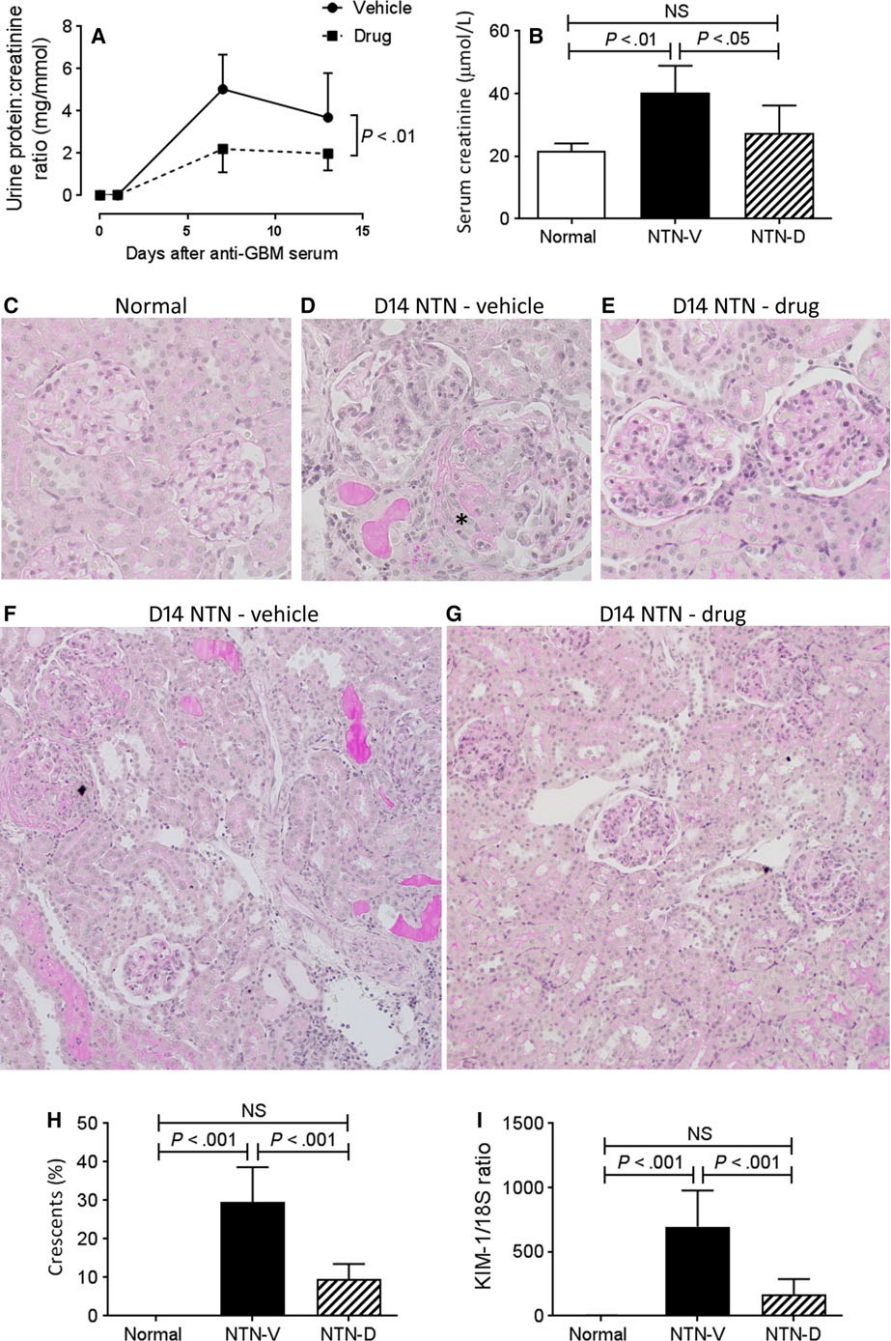
Kidney injury in day 14 nephrotoxic serum nephritis (NTN). Disease was induced in groups of 8 WKY rats which were treated with GS‐444217 (drug; NTN‐D) or vehicle (NTN‐V) alone. A group of 6 normal rats served as control. A, Urine total protein:creatinine ratio. B, Serum creatinine. C‐G, periodic acid Schiff stained sections: C, normal rat kidney; D, vehicle‐treated disease exhibiting hypercellularity, crescent formation (*) and thrombosis; E, drug‐treated disease showing hypercellularity; F, vehicle‐treated disease showing extensive tubulointerstitial damage with cast formation, tubular dilation and inflammatory cell infiltration: G, drug‐treated disease showing mild tubulointerstitial damage. H, Percentage of glomeruli exhibiting crescent formation. I, RT‐PCR analysis of mRNA levels for the tubular damage marker, KIM‐1. Original magnification, ×400 (C‐G), ×200 (F,G). Data are mean ± SD. A, analysed by repeated measures two‐way ANOVA; B,H,I, analysed by one‐way ANOVA with Tukey's multiple comparison test

**Figure 4:**
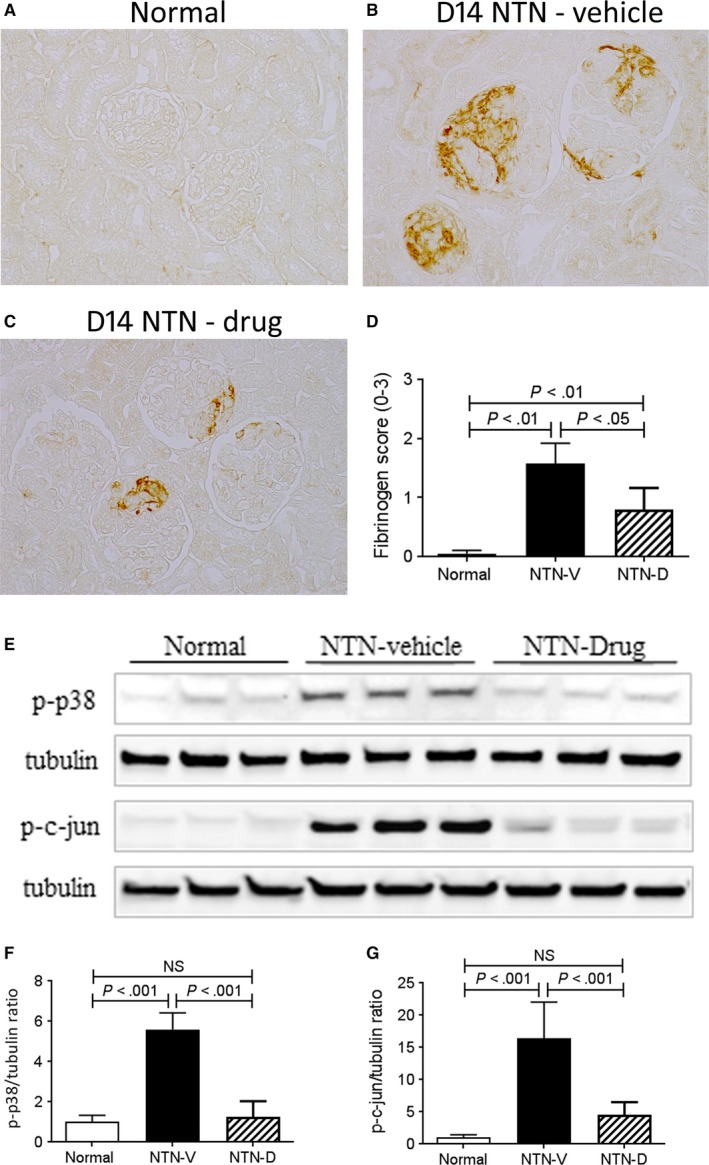
Glomerular fibrin deposition and kidney p38 and c‐Jun amino terminal kinase activation in day 14 nephrotoxic serum nephritis (NTN). Disease was induced in groups of 8 WKY rats which were treated with GS‐444217 (drug; NTN‐D) or vehicle (NTN‐V) alone. A‐C, Immunostaining for fibrinogen: A, little staining is seen in normal rat kidney; B, pronounced fibrin deposition is seen in the glomerular tuft and in crescents in vehicle‐treated disease, (C) drug treatment partially reduces fibrin deposition in day 14 NTN. D, Quantification of the glomerular fibrin staining score (0‐3+). E, Western blot analysis of whole kidney lysates for p‐p38 and p‐c‐Jun with tubulin reprobe. Graphs show quantification for p‐p38 (F) and p‐c‐Jun (G). Data are mean ± SD. D, Analysis by nonparametric one‐way ANOVA with Dunn's multiple comparison test; F,G, analysis by one‐way ANOVA with Tukey's multiple comparison test

To investigate whether ASK1 inhibition can prevent the development of progressive crescentic glomerulonephritis, we examined the NTN model in susceptible inbred WKY rats.^31^, ^32^ Compared to SD rats, induction of NTN in WKY rats causes less severe glomerular injury on day 1, but WKY rats reliably develop crescentic lesions by day 14.^31^, ^32^ Induction of NTN in vehicle‐treated WKY rats resulted in proteinuria on days 7 and 14, together with significant renal function impairment on day 14 (Figure 3A,B). This was associated with marked glomerular damage on day 14 NTN featuring hypercellularity, thrombosis, atrophy and crescent formation (Figure 3D). Crescent formation was evident in 30% of glomeruli (Figure 3H), while fibrin deposition was evident in the glomerular tuft and in Bowman's space (Figure 4A‐D). Secondary tubulointerstitial damage was evident in vehicle treated animals on day 14 NTN with patchy areas of tubular dilatation, atrophy, cast formation and interstitial cell infiltration (Figure 3F). Consistent with histologic tubular damage, mRNA levels of the tubular damage marker KIM‐1 were markedly elevated in vehicle‐treated day 14 NTN (Figure 3I).

Drug treatment significantly reduced the severity of proteinuria and prevented renal impairment on day 14 NTN (Figure 3A,B). Glomerular damage was substantially attenuated, with a 66% reduction in crescent formation, as well as a significant reduction in glomerular fibrin deposition (Figures 3E,H,4A‐D). Drug treated rats also showed attenuation of tubulointerstitial damage based on histology and KIM‐1 expression (Figure 3G,I). Drug treatment did not modify glomerular deposition of sheep IgG, rat IgG or rat C3 (Figure S1), indicating that GG‐444217 had no effect on the immune response to sheep IgG, consistent with the fact that drug treatment did not begin until 7 days after immunization with sheep IgG when the T‐ and B‐cell response is fully established.

Activation of p38 and JNK signalling was evident in vehicle‐treated day 14 NTN as shown by Western blotting of whole kidney samples (Figure 4E‐G). Drug treatment efficiently suppressed p38 activation and substantially reduced JNK activation (Figure 4E‐G).

**Figure 5:**
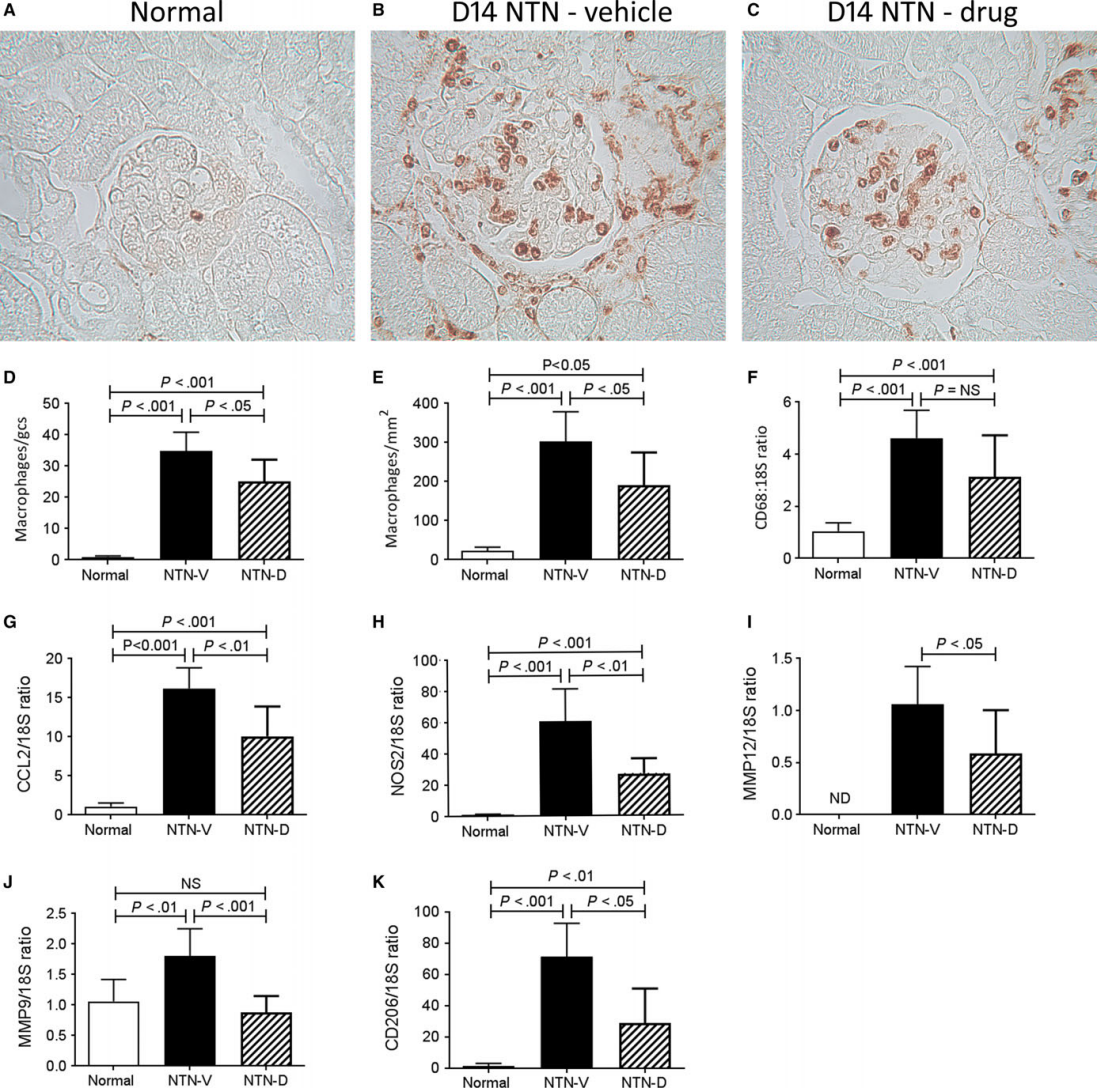
Macrophage recruitment and inflammation in day 14 nephrotoxic serum nephritis (NTN). Disease was induced in groups of 8 WKY rats which were treated with GS‐444217 (drug; NTN‐D) or vehicle (NTN‐V) alone. A‐C, Immunostaining for ED1+ macrophages: A, normal rat kidney; B, vehicle‐treated disease shows prominent macrophage accumulation in glomeruli and through the interstitium; C, drug treatment also has substantial glomerular and interstitial macrophage accumulation. Quantification of the number of ED1+ macrophages in: D, glomeruli, and (E) the interstitium. RT‐PCR analysis of mRNA levels for: F, CD68; G, CCL2; H, NOS2; I, MMP‐12; J, MMP‐9, and; K, CD206. Data are mean ± SD. Analysis by one‐way ANOVA with Tukey's multiple comparison test

A prominent glomerular and interstitial infiltrate of CD68+ macrophages was evident on day 14 NTN in vehicle‐treated animals (Figure 5A‐E). Analysis of whole kidney tissue showed an increase in mRNA levels for CD68 and for the monocyte chemokine CCL2 (Figure 5F,G). RT‐PCR analysis identified a substantial increase in both M1‐type (NOS2, MMP‐9 and MMP‐12) and M2‐type (CD206) macrophage markers (Figure 5H‐K). Drug treatment resulted in a small, but significant, reduction in both glomerular and interstitial macrophage accumulation (Figure 5C‐E). This reduction in macrophage accumulation was associated with reduced levels of the monocyte chemokine CCL2 and a substantial reduction in expression of both M1 and M2 macrophage markers (Figure 5G‐K).

**Figure 6:**
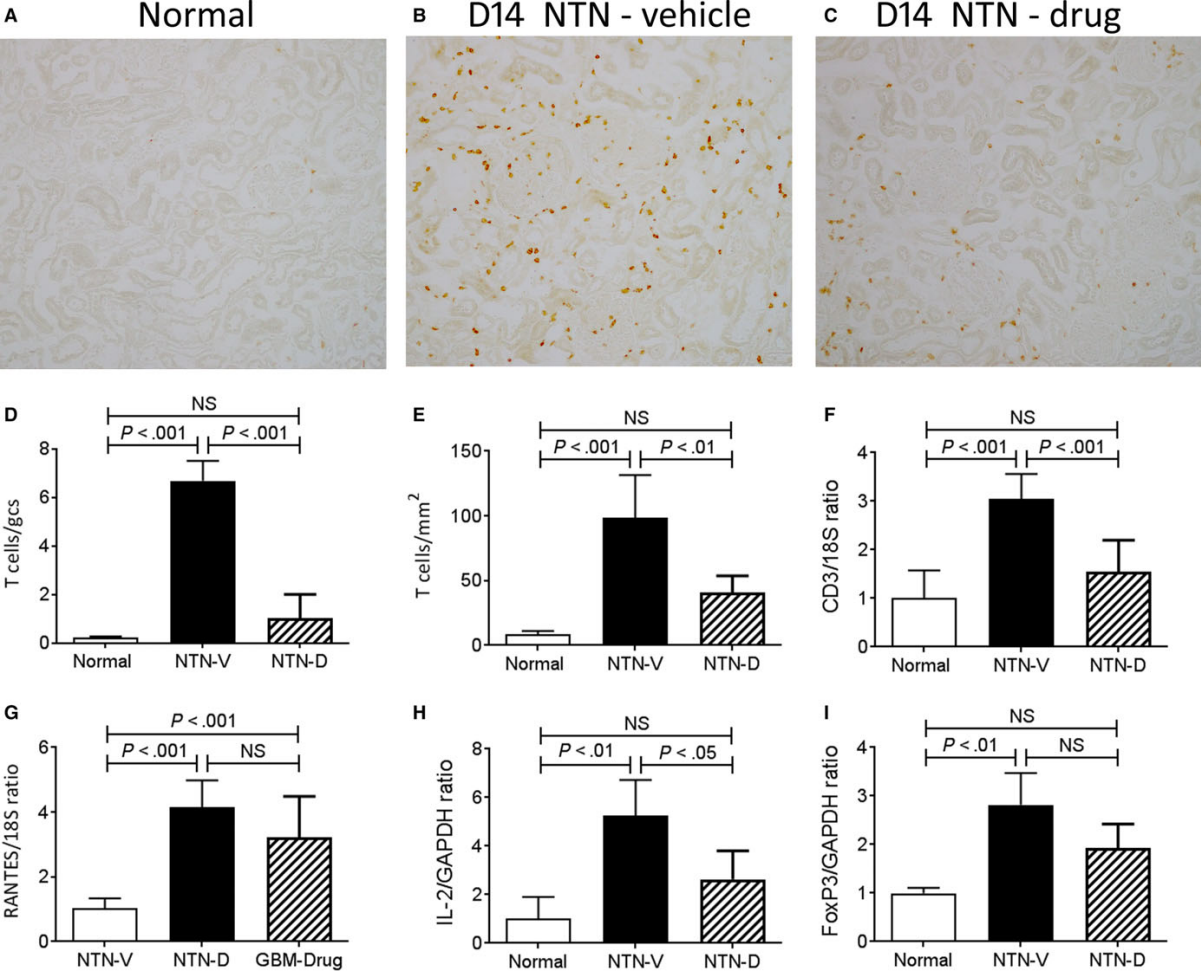
T‐cell infiltration in day 14 nephrotoxic serum nephritis (NTN). Disease was induced in groups of 8 WKY rats which were treated with GS‐444217 (drug; NTN‐D) or vehicle (NTN‐V) alone. A‐C, Immunostaining for R73+ T cells: A, normal rat kidney; B, vehicle treated disease shows a substantial infiltrate of T cells in glomeruli and the interstitium; C, drug treatment shows a marked reduction in T‐cell infiltration in both compartments. Quantification of the number of R73+ T cells in: D, glomeruli, and (E) the interstitium. RT‐PCR analysis of mRNA levels for: F, CD3; G, CCL5; H, IL‐2, and; I, FoxP3. Data are mean ± SD. Analysis by one‐way ANOVA with Tukey's multiple comparison test

Immunostaining identified a glomerular and interstitial T‐cell infiltrate on day 14 NTN (Figure 6A‐E). Analysis of whole kidney by RT‐PCR showed increased T‐cell infiltration (CD3ε), increased expression of the T‐cell chemokine CCL5 together with T‐cell activation (IL‐2) and infiltration of regulatory T cells (FoxP3) (Figure 6F‐I). Drug treatment markedly reduced the glomerular T‐cell infiltrate and reduced interstitial T‐cell infiltration, with a significant reduction in CD3ε mRNA levels (Figure 6A‐F). Consistent with these findings, IL‐2 mRNA levels were reduced (Figure 6H), whereas mRNA levels of CCL5 and FoxP3 expression were not significantly affected by drug treatment (Figure 6G,I).

**Figure 7:**
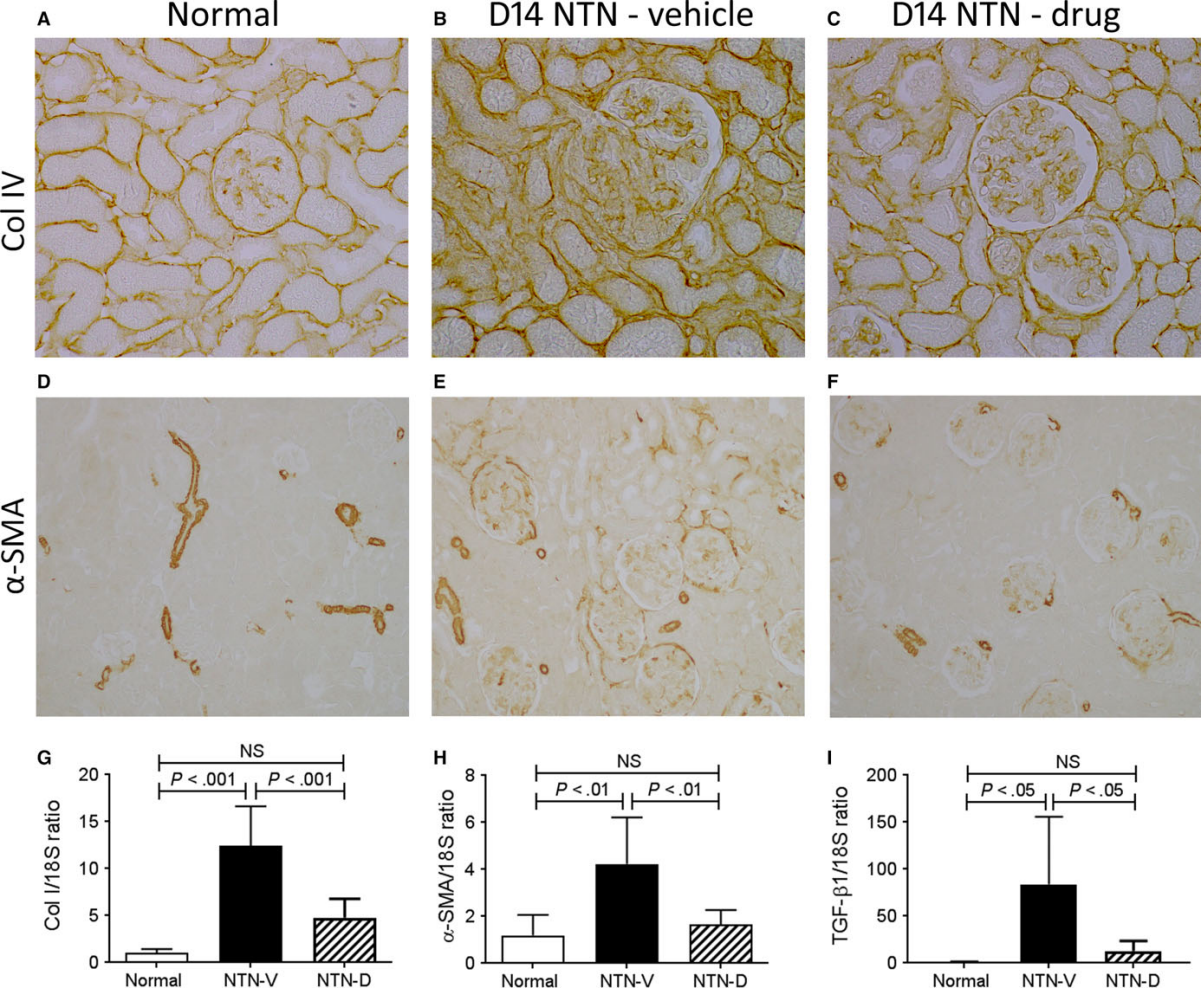
Renal fibrosis in day 14 nephrotoxic serum nephritis (NTN). Disease was induced in groups of 8 WKY rats which were treated with GS‐444217 (drug; NTN‐D) or vehicle (NTN‐V) alone. A‐C, Immunostaining for collagen IV: A, normal rat kidney shows collagen IV in the glomerular and tubular basement membranes; B, vehicle‐treated disease shows increased deposition of collagen IV in both glomeruli and the interstitium; C, drug treatment reduces shows a mild increase in collagen IV staining compared to normal. D‐F, Immunostaining for α‐SMA: D, normal rat kidney shows α‐SMA staining in vessel walls; E, vehicle‐treated disease shows α‐SMA+ myofibroblasts within glomeruli, in the periglomerular area and in the interstitium, and; F, drug‐treated disease shows only small numbers of α‐SMA+ cells in glomeruli and the interstitium. RT‐PCR analysis of mRNA levels for: G, collagen I; H, α‐SMA; and; I, TGF‐β1. Data are mean ± SD. Analysis by one‐way ANOVA with Tukey's multiple comparison test

Vehicle‐treated rats developed significant glomerulosclerosis and interstitial fibrosis on day 14 NTN as shown by increased deposition of collagen IV and the appearance of α‐SMA+ myofibroblasts in the glomerular tuft, around Bowman's capsule and within the interstitium (Figure 7A‐E). Glomerular and interstitial fibrosis was substantially reduced by drug treatment (Figure 7A‐F). This suppression of renal fibrosis was associated with reductions in mRNA levels of collagen I, α‐SMA and TGF‐β1 (Figure 7G‐I).

## 4 DISCUSSION

The current study demonstrated that the ASK1 inhibitor, GS‐444217, is effective in suppressing activation of p38 and, to a lesser degree, JNK pathways in the acute and progressive phases of rat models of NTN. This extends previous studies in which *Ask1* gene deletion or GS‐444217 treatment identified that ASK1 signalling is required for pathologic activation of p38 and, to a lesser degree, JNK pathways in the mouse obstructed kidney and in mouse diabetic nephropathy.^23^, ^24^ Thus, in three distinct models of renal injury induced by diverse insults (diabetes, mechanical stretch and immunity), it is possible that oxidative stress‐induced activation of ASK1 is a common mechanism for activation of p38/JNK signalling. One limitation of this study is the lack of commercial antibodies suitable for directly detecting ASK1 activation in tissue samples. Thus, we can only infer that GS‐444217 suppressed renal injury in the NTN model via acting on ASK1. However, this is a reasonable conclusion to draw given the exquisite selectivity of GS‐444217 for targeting ASK1 and that studies in the unilateral ureteric obstruction model of renal fibrosis gave remarkably similar results using *Ask1* gene deletion and GS‐444217 treatment (23 and unpublished data).

Suppression of the acute and progressive phases of rat NTN by treatment with GS‐444217 treatment is attributed to inhibition of p38 and JNK signalling pathways. This conclusion is based on three points: GS‐444217 treatment substantially inhibited activation of the p38 and JNK pathways; GS‐444217 treatment gave similar results to that seen with individual blockade of p38 or JNK pathways; and p38 and JNK are the only terminal targets of ASK1 activation thus far identified. A comparison of the effects of GS‐444217 treatment to that previously described with inhibitors of p38 or JNK enzymes is considered below.

The inhibition of acute glomerular thrombosis, proteinuria and glomerular up‐regulation of TNF‐α expression seen with GS‐444217 on day 1 NTN in SD rats is consistent with the protection evident with p38 or JNK inhibition. Blockade of p38 reduced acute neutrophil infiltration and thrombosis with a partial reduction in proteinuria on day 1 NTN,^10^ while JNK inhibition reduced proteinuria and suppressed markers of inflammation (TNF‐α, CCL2 and NOS2) on day 1 NTN.^16^ Indeed, adoptive transfer studies have shown that macrophages can cause acute glomerular injury in the rat NTN model,^33^ and that this is dependent upon JNK activation within the infiltrating macrophages.^34^ GS‐444217 treatment did not reduce glomerular macrophage infiltrate on day 1 NTN, but did inhibit macrophage activation based on suppression of TNF‐α and NOS2 mRNA levels.

The development of glomerular crescents, renal fibrosis and renal impairment is characteristic features in the progression of the rat NTN model.^29^, ^35^ The ability of GS‐444217 treatment to reduce proteinuria, crescent formation, renal fibrosis and renal impairment in this model is consistent with studies employing p38 or JNK inhibitors.^8^, ^9^, ^11^, ^16^, ^17^ Fibrin deposition plays a role in crescent formation in this model,^36^ and ASK1/p38 signalling can promote platelet activation in response to pathologic stimuli.^37^ Consistent with these findings, both GS‐444217 treatment and p38 inhibition significantly reduced glomerular fibrin deposition and crescent formation in rat NTN.^11^ Macrophages are essential for crescent formation in the NTN model.^31^, ^38^ Blockade of p38 suppressed glomerular macrophage recruitment and renal expression of the monocyte/macrophage chemokine, CCL2, in association with reduced crescent formation.^8^, ^9^, ^11^ In contrast, JNK blockade inhibits macrophage activation rather than recruitment while protecting against the development of glomerular crescents and renal impairment in the NTN model.^16^, ^17^ Consistent with these studies, we found that GS‐444217 treatment reduced macrophage recruitment and macrophage activation. In particular, expression of MMP‐9 and MMP‐12; enzymes known to facilitate macrophage‐mediated renal injury and crescent formation in the NTN model,^39^, ^40^, ^41^ was reduced by GS‐444217. Furthermore, inhibition of pro‐inflammatory and pro‐fibrotic molecule expression by GS‐444217 is likely to be a combination of direct and indirect effects. In vitro studies show a direct role for JNK and p38 signalling in up‐regulation of both pro‐inflammatory (eg, TNF‐α, NOS2, MCP‐1, MMP‐12) and pro‐fibrotic (eg, Col I, α‐SMA and TGF‐β1) molecules.^6^, ^17^, ^23^, ^34^ However, while GS‐444217‐induced suppression of pro‐inflammatory molecule expression is likely to be a direct effect of p38/JNK blockade—especially on day 1 NTN—the reduction in pro‐fibrotic molecule expression is most probably an indirect, secondary effect of suppressing renal inflammation.

T cells play an important role in crescent development in the rat NTN model.^42^ JNK inhibition does not affect glomerular T‐cell infiltration in the rat NTN model,^17^ while this has not been investigated in studies of p38 blockade in this model. GS‐444217 treatment caused a profound inhibition of glomerular T‐cell infiltration which is presumably because of inhibition of p38 signalling. The chemokine CCL5 can promote T‐cell recruitment in the NTN model,^43^ but GS‐444217 did not affect CCL5 mRNA levels. Inhibition of T‐cell infiltration may have contributed to the protective effect of GS‐444217 treatment in this model or it could be an indirect effect of suppressing renal injury. This requires further investigation.

Animal data combined with human biopsy studies have identified the p38 and JNK signalling pathways as potential therapeutic targets.^22^ However, both p38 and JNK inhibitors have failed in clinical trials of nonrenal diseases, in part because of toxicity issues, indicating that complete blockade of p38 or JNK signalling is not desirable.^19^, ^20^ In contrast to the foetal lethal phenotype of mice lacking *p38* or *Jnk1/2* genes, mice lacking *Ask1* have a normal phenotype arguing that ASK1 function is not required for normal homoeostatic mechanisms.^3^, ^22^ Indeed, the requirement for oxidative stress in ASK1‐dependent activation of p38/JNK signalling means that, at least in theory, blocking ASK1 will prevent p38/JNK signalling in some pathologic conditions but should have little effect upon physiological p38/JNK signalling and thereby limit toxicity. ASK1 inhibitor treatment has been reported to be safe and efficacious to reduce liver fibrosis in a phase II trial of nonalcoholic steatohepatitis,^44^ while the results of a trial evaluating ASK1 inhibitor treatment in patients with diabetic kidney disease have yet to be reported.

In conclusion, this study demonstrates that administration of an ASK1 inhibitor suppressed activation of the p38 and, to a lesser extent, the JNK signalling pathway in a rat model of crescent glomerulonephritis. ASK1 inhibitor treatment was effective in suppressing both acute glomerular injury and the development of crescentic disease. ASK1 blockade is a potential therapeutic strategy in rapidly progressive glomerulonephritis.

## CONFLICT OF INTEREST

D.G.B. and J.T.L. are employees of Gilead Sciences. D.J.N.‐P. has previously received funding from Gilead for studies of ASK1.
