# Supplementary material for: ASK1 inhibitor treatment suppresses p38/JNK signalling with reduced kidney inflammation and fibrosis in rat crescentic glomerulonephritis
Source: J Cell Mol Med. 2018 Jul 11;22(9):4522–33. doi: 10.1111/jcmm.13705 (PMC6111820; doi:10.1111/jcmm.13705)
Supplement: Supplementary file 1 [file JCMM-22-4522-s001.pdf]

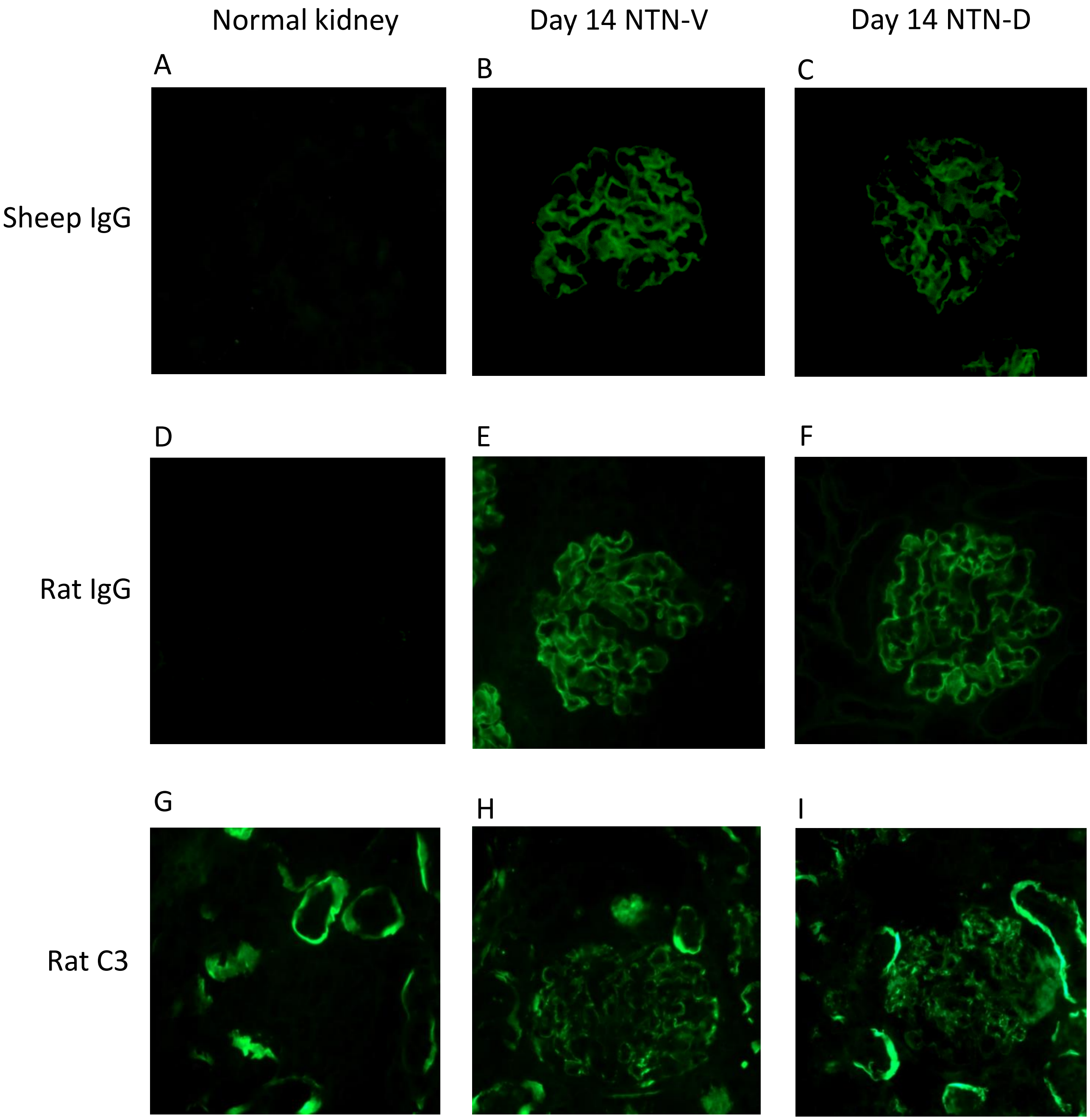

Supplementary Figure 1. Glomerular deposition of immune reactants in day 14 nephrotoxic serum nephritis (NTN). (A-C) Immunostaining for sheep IgG; (A) sheep IgG is absent in normal rat kidney, while (B) vehicle treated disease (NTN-V) and (C) GS-444217 treated disease (drug; NTN-D), show an equivalent intensity of linear sheep IgG deposition along the glomerular basement membrane. (D-F) Immunostaining for rat IgG; (D) rat IgG is absent in normal rat kidney, while (E) vehicle treated disease (NTN-V) and (F) GS-444217 treated disease (drug; NTN-D), show an equivalent intensity of linear rat IgG deposition along the glomerular basement membrane. (G-I) Immunostaining for rat C3; (G) C3 is present in proximal tubules but absent from the glomerulus in normal rat kidney, while (H) vehicle treated disease (NTN-V) and (I) GS-444217 treated disease (drug; NTN-D), show an equivalent deposition of rat C3 in glomeruli.
